# Characterization of oral and cloacal microbial communities in cold-stunned Kemp’s ridley sea turtles (*Lepidochelys kempii*) during the time course of rehabilitation

**DOI:** 10.1371/journal.pone.0252086

**Published:** 2021-05-27

**Authors:** Kerry L. McNally, Charles J. Innis, Adam Kennedy, Jennifer L. Bowen

**Affiliations:** 1 Animal Health Department, New England Aquarium, Boston, Massachusetts, United States of America; 2 University of Massachusetts, Boston, Massachusetts, United States of America; 3 Rescue & Rehabilitation Department, New England Aquarium, Boston, Massachusetts, United States of America; 4 Department of Marine and Environmental Sciences, Marine Science Center, Northeastern University, Nahant, Massachusetts, United States of America; University of Illinois College of Medicine, UNITED STATES

## Abstract

Microbial communities of animals play a role in health and disease, including immunocompromised conditions. In the northeastern United States, cold-stunning events often cause endangered Kemp’s ridley turtles (*Lepidochelys kempii*) to become stranded on beaches in autumn. These sea turtles are admitted to rehabilitation facilities when rescued alive and are presumed immunocompromised secondary to hypothermia. To better understand the role that microbes play in the health of cold-stunned sea turtles, we characterized the oral and cloacal microbiome from Kemp’s ridley turtles at multiple timepoints during rehabilitation, from admission to pre-release, by using Illumina sequencing to analyze the 16S rRNA gene. Microbial communities were distinct between body sites and among turtles that survived and those that died. We found that clinical parameters such as presence of pneumonia or values for various blood analytes did not correlate with oral or cloacal microbial community composition. We also investigated the effect of antibiotics on the microbiome during rehabilitation and prior to release and found that the type of antibiotic altered the microbial community composition, yet overall taxonomic diversity remained the same. The microbiome of cold-stunned Kemp’s ridley turtles gradually changed through the course of rehabilitation with environment, antibiotics, and disease status all playing a role in those changes and ultimately the release status of the turtles.

## Introduction

Kemp’s ridley turtles (*Lepidochelys kempii*) are listed as Critically Endangered by the International Union for the Conservation of Nature [1]. The species faces global challenges due to fisheries interactions, legal and illegal harvest, habitat loss, pollution, vessel strike, and climate change [2–4]. In addition to anthropogenic causes of population decline, sea turtles are also susceptible to several diseases and presumed immunocompromising conditions that require rehabilitation and medical intervention [3,5]. One example of this is cold-stunning, or hypothermia. Cold-stunning occurs when turtles are exposed to water temperatures below 10°C [3,5–7]. Large cold-stunning events involving juvenile Kemp’s ridley, green (*Chelonia mydas*), and loggerhead turtles (*Caretta caretta*) occur annually in Massachusetts when turtles do not migrate south before water temperatures drop during autumn [7,8]. Cold-stunned turtles cease swimming and may become stranded on beaches when forced ashore by tidal activity and wind. Warming sea water temperatures are predicted to cause a continued trend of increasing numbers of stranded Kemp’s ridley turtles by increasing the distribution of turtles to the northeastern United States and creating a bridge from the Gulf Stream to nearshore waters [9]. Common sequelae resulting from chronic cold-stunning include cardiorespiratory depression, dehydration, reduced renal function, pneumonia, sepsis, osteomyelitis, and death [10–17]. Kemp’s ridley turtles comprise the majority of turtles that strand each year in the northeastern US, and when found alive, they are transported to wildlife hospitals, such as the New England Aquarium (NEAq), for medical care. NEAq is the primary rehabilitation center for sea turtles stranded in Massachusetts, where turtles are triaged and rehabilitated over several months until released or transported to secondary facilities for continued care. Affected turtles often require intensive medical management over several months of hospitalization, during which they are serially evaluated by physical examinations, hematology and plasma biochemical evaluations, radiography, and other methods needed to guide their recovery [3,5]. It is not known whether cold-stunned turtles’ microbial communities are affected during rehabilitation, but it is possible that medical management and the captive environment could lead to a dysbiosis, or shift in the microbiome of the affected turtles.

Chronic disease conditions or environmental stressors can cause dysbiosis in humans and other animals, but there is limited information on sea turtle microbiomes [18–21]. Research from other species reveals that these chronic diseases may not be caused by a single agent, but rather by dysbiosis of the microbial communities that play a role in health and immunity [21–23]. Perturbation of natural microbial communities drives many chronic diseases in humans, including gut, oral, skin, and lung disorders; thus, understanding dysbiosis may improve diagnostic and therapeutic management [23]. Further, dysbiosis of one body site may affect other sites because of microbial communities’ effect on the host immune system. For example, the gut microbiome plays a role in host immune system function, directly influencing diseases of the gut, but also affecting other sites such as the nervous system or respiratory tract [22].

One conceptualization of dysbiosis is that healthy microbiomes are similar, but a dysbiotic or unhealthy microbiome is each altered in a unique way [21,24]. Healthy microbiomes have little variability, while stressed or diseased microbiomes have a wider range of altered compositions compared to the healthy microbiomes [21]. For example, frogs infected with the fungus, *Batrachochytrium dendrobatidis* (Bd) showed more variability in their skin microbiome than frogs that were not infected [25]. Thus, it is important to understand whether microbial community response to disease states is stochastic, or if it is driven by deterministic processes triggered by environmental surroundings [21]. Diseases of corals, such as black band disease and white-plague disease, are associated with variable shifts in microbial communities, including increases in opportunistic pathogens, polymicrobial infection, reduction in commensal bacteria, or enrichment of bacteria with pathogenic potential [23,26]. Many stressors may lead to dysbiosis of sea turtles during rehabilitation, including the initial cold-stunned event, stress of rehabilitation, antibiotic treatment, and captive diet.

Thus far, insights into the microbiome of sea turtles primarily focused on the cloacal or fecal microbiome of loggerhead and green turtles [27–32] or only focused on adult nesting female turtles of all species [33]. The fecal microbiome was distinct between wild-captured green turtles and stranded green turtles, with stranded animals having a higher proportion of Proteobacteria, specifically Gammaproteobacteria, compared to wild turtles that had feces dominated by Firmicutes [28]. Rehabilitation also appears to affect the microbiome. The cloacal microbial communities differed before and after rehabilitation of green turtles, with post rehabilitation turtles having more similar microbiomes due to both environmental effects and controlled diet during hospitalization [29]. Green turtles in rehabilitation also have shifts in their microbiome attributed to receiving a high protein diet during recovery [34]. Length of rehabilitation and antibiotic exposure also affects the fecal microbiome of Kemp’s ridley turtles in rehabilitation [35].

During rehabilitation for cold-stunning, Kemp’s ridley turtles are treated with antibiotics to prevent or treat secondary infections [36,37]. Although antibiotics are important to treating infectious diseases, these medications interact with entire microbial communities, which can affect immune homeostasis of the host and potentially lead to dysbiosis [38–40]. In humans, even a short term course of antibiotic treatment can have a long lasting impact. For example, the microbiome of the throat and gut became altered after just one week of treatment with clarithromycin and metronidazole, and the microbiome remained perturbed, in some cases, for up to four years after treatment [41]. Different antibiotics have different effects on the shift in microbial community assemblage, but all generally result in a decrease in diversity of the microbial community, coupled with varying timeframes until the community returns to the pretreatment state [38]. Exposure to antibiotics can also lead to antibiotic resistance due to increases in antibiotic resistance genes in the microbial community [41–43]. Understanding the effect that antibiotic treatment, and the rehabilitation process more broadly, has on the microbiome of Kemp’s ridley turtles is important for optimizing their chances for success once they are reintroduced to the wild.

In this study, we investigated the microbial communities of cold-stunned Kemp’s ridley turtles through the time course of rehabilitation at NEAq. Our first objective was to characterize the oral and cloacal microbiome of the cold-stunned turtles. We hypothesized that there was a distinct and diverse microbial community at each body site. Second, we identified bacteria that were associated with mortality versus survival and we evaluated the microbiome at admission to determine if there were correlations with clinical variables such as hematologic parameters (i.e. complete blood counts) or disease status (i.e. pneumonia). We also evaluated temporal effects of rehabilitation to determine alterations to the turtle microbiome from the time they were admitted to the hospital through the end of their hospitalization. We hypothesized that the microbiome of turtles at intake (i.e. directly from the wild) would shift when cold-stunned turtles were hospitalized, brought to appropriate body temperatures, and medically managed. We also hypothesized that antibiotics would alter microbiomes compared to turtles that were not administered antibiotics and that the microbial community assemblages would converge toward that of the turtles that did not receive antibiotics once they were considered clinically healthy (i.e. after discontinuing antibiotics and prior to release).

## Materials and methods

### Ethics statement

This study was approved by the NEAq Institutional Animal Care and Use Committee (Protocol #2015–16) and conducted under the US Department of the Interior Fish and Wildlife Service Permit# TE-697823.

### Sample collection

We collected oral and cloacal samples from juvenile Kemp’s ridley turtles admitted to NEAq during the 2015 cold-stun event (November and December 2015). We chose turtles at random to be enrolled in the study and to have radiographs taken prior to their intake exam to assess the degree of lung abnormalities. The attending veterinarian categorized the turtle as pneumonia or not based on their interpretation of the radiographs, hereafter referred to as Pneumonia or Non-Pneumonia turtles [44,45]. We collected blood during physical exams from the dorsal cervical sinus and analyzed it immediately using a blood gas and biochemical analyzer (pHOx Ultra, NOVA Biomedical, Waltham, MA). Since the analyzer generates data for blood at 37°C, we used the patient’s body temperature to perform previously published calculations to mathematically correct for temperature-dependent variables, including pH, partial pressure of carbon dioxide, and partial pressure of oxygen, and we calculated ionized calcium with the temperature corrected pH [10,46–49]. We calculated bicarbonate using the Henderson-Hasselbalch equation [47,50]. At day three of rehabilitation, additional blood samples were collected and transported to a commercial veterinary diagnostic laboratory (IDEXX Laboratories, North Grafton, MA) where a complete blood count and chemistry panel was performed [12].

Prior to the intake physical exam on day 0, we collected samples for microbiome analysis from the oral cavity and cloaca of each animal. We took an oral swab by gently swabbing the glottis of the turtle with a sterile cotton tipped applicator, using a sanitized bite block (Nylabone Products, Neptune City, NJ, USA) to keep the mouth open. We then took a cloacal swab by inserting a cotton tipped applicator gently into the cloaca approximately 2.5 cm and swabbing the mucosa. We placed swabs into individual cryovials and immediately placed them on dry ice after collection, then moved them to an ultra-low freezer (-80°C) within 15 minutes for later DNA extraction and sequencing.

**Fig 1:**
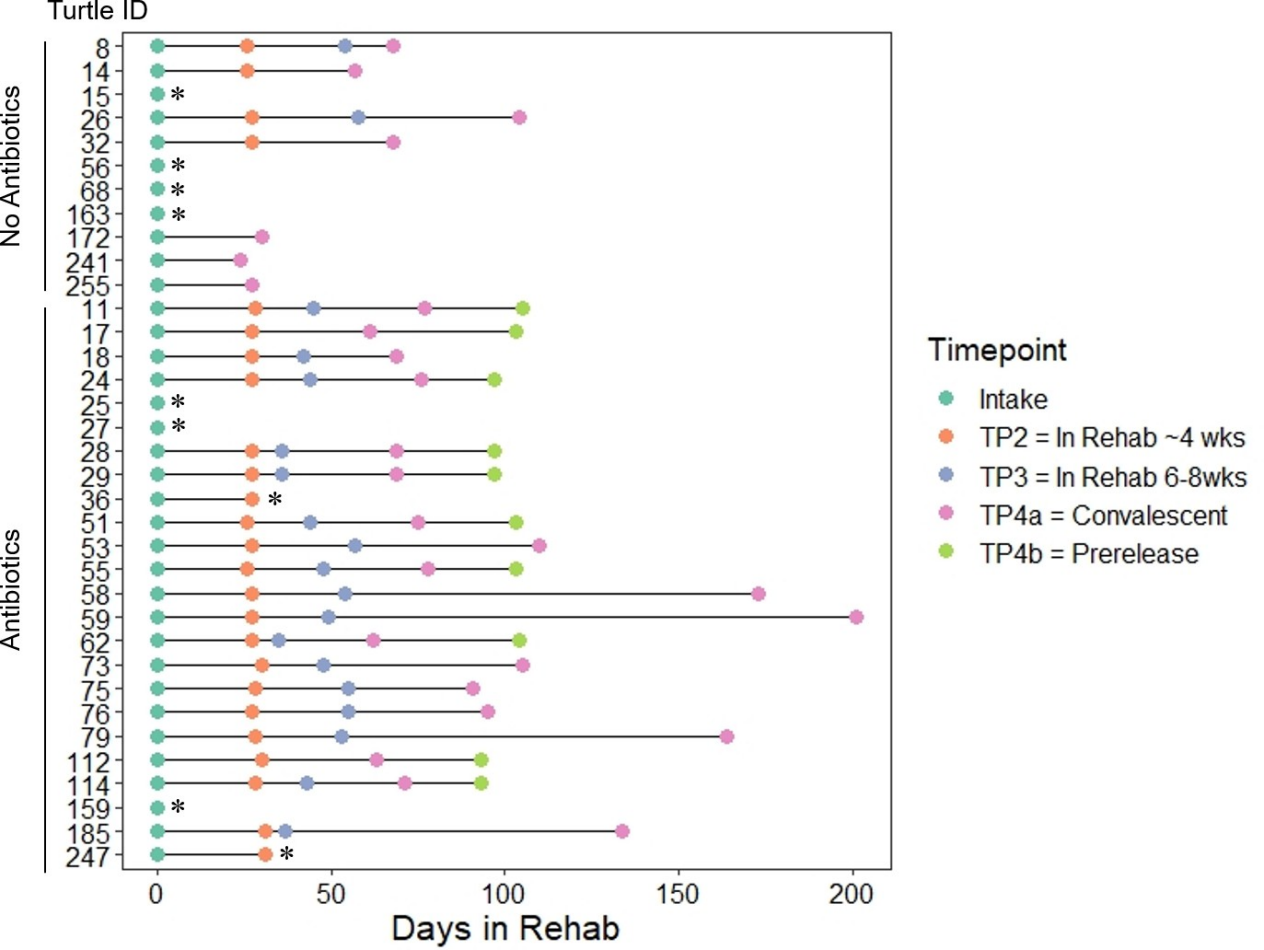
Oral and cloacal samples collected through rehabilitation. Swabs collected for each individual turtle (y axis) during rehabilitation. Each color denotes the timepoint in rehab. Convalescence (TP4a) is when the turtle is considered clinically healthy. Prerelease (TP4b) was only collected on turtles that remained in the hospital >30 days after TP4a was collected. Whether antibiotics were received or not is indicated on the y axis. * indicates that the turtle died after that sample was collected.

NEAq veterinarians prescribed antibiotics (ceftazidime 22 mg/kg intramuscularly every 3 days or oxytetracycline 42 mg/kg subcutaneously every 6 days) for the turtles, as necessary, based on radiograph findings and blood analysis [36,51]. Selection of the initial antibiotic was based on an unrelated study, with ~50% of the turtles receiving one of the two drugs. In some cases, additional antibiotics or a change in antibiotic was prescribed later in rehabilitation based on clinical needs. We sampled the surviving turtles throughout rehabilitation at timepoints dependent on clinical status. We collected oral and cloacal swabs at a second timepoint, four weeks after the turtles were admitted. Timepoint 3 was slated to be at eight weeks after admittance but, in some cases, was conducted as early as six weeks to ensure that the sample was collected prior to the discontinuance of antibiotics. Timepoint 4a was collected when the turtle was classified as convalescent, or clinically healthy, which was defined as 30 days after antibiotics were discontinued. If a turtle was not on antibiotics, convalescence was determined based on when the animal was ready for release, which depended on appetite, physical exam, and transport preparation. We collected oral and cloacal swabs at an additional timepoint, timepoint 4b, prior to release if the turtle remained at NEAq more than 4 weeks after timepoint 4a was collected. Overall, turtles in the longitudinal study received 2 to 5 oral and cloacal swabs during their time in rehabilitation, except those that died after intake sampling (Fig 1). During rehabilitation, turtles were maintained in tanks of filtered saltwater at approximately 24°C and they were offered food items of herring and squid once to twice daily.

### DNA extraction

We extracted DNA from the swabs using a phenol:chloroform:isoamyl extraction protocol adapted from Mettel et al. [52]. We first suspended the swabs in 700 μL of PBL lysis buffer (6% water saturated phenol, 5 mM disodium EDTA, 0.1% (wt/vol) sodium dodecyl sulfate, 5 mM Tris HCL, pH 5.7) by vortexing for 5 minutes and centrifuging for 2 minutes at 17,000 x g. We removed the supernatant and placed it in a clean 2 mL tube. After removal of the supernatant, we added 700 μL TPM buffer (50 mM Tris, pH 7.0, 1.7% (wt/vol) polyvinyl pyrrolidone, and 20 mM MgCl_2_) to the swab; after vortexing for 1 minute and centrifuging for 2 minutes at 17,000 x g, we then added the second supernatant to the tube with the first supernatant. We supplemented the combined supernatant with 800 μL of a phenol:chloroform:isoamyl alcohol solution (pH 6.7+, 25:24:1) and centrifuged for 7 minutes at 17,000 x g. We transferred the upper aqueous layer to a sterile tube and added 0.7 volumes of 100% isopropanol and 0.1 volumes of 3 M sodium acetate. After centrifugation for 4 minutes at 17,000 x g, the supernatant was decanted, we washed the pellet with 70% ethanol, and allowed it to air dry. We then resuspended the dried pellet in 50 μL nuclease-free water and stored it at -80°C until amplification. We verified all DNA extracts by gel electrophoresis, including negative controls of unused sterile swabs, to ensure there was no contamination from supplies and solutions used in the extraction.

After verification, we amplified DNA extracts in triplicate using bacterial specific (515F and 806R), uniquely barcoded, 16S rRNA gene primers containing adaptors for Illumina sequencing [53]. Each 25 μL PCR reaction contained 12.5 μL Phusion Master Mix (ThermoFisher), 0.5 μL primers, 11 μL diethylpyrocarbonate (DEPC) water, and 1 μL of DNA. Thermal cycling conditions using S1000 thermal cycler (Bio-Rad, Hercules, CA, USA) had an initial denaturation at 98°C for 3 min, then 40 cycles consisting of 45 seconds at 98°C, 60 seconds at 51°C, and 90 seconds at 72°C. The triplicate PCR product was verified via gel electrophoresis, and we excised the target bands and purified them using the QIAquick PCR Purification Kit (QIAGEN, Valencia, CA, USA) following the manufacturer’s protocols. We then quantified the purified product using a Qubit 2.0 Fluorometer (ThermoFisher, Waltham, MA, USA) and pooled all samples together in equimolar concentrations. Sequencing was performed on the Illumina MiSeq platform with a paired-end V2 300 cycle kit at the University of Massachusetts, Boston.

Any samples that had poor sequencing read quality or low sequencing depth were reamplified in triplicate as described above. However, for these samples we purified the resulting PCR product using AMPure XP (Beckman Coulter, Inc. Indianapolis, IN, USA) following manufacturers guidelines using the 0.8:1.0 ratio of bead-to-sample to target 300 bp and above. After purification, we quantified the DNA using the Agilent D1000 ScreenTape System (Agilent Technologies, Inc, Waldbronn, Germany) following manufacturers guidelines for more precise quantification and band size visualization. We pooled the purified PCR product to equimolar concentration based on the concentration of the desired band size range. We used a BluePippin™ (Sage Science Inc., Beverly, MA, USA), following manufacturer’s instructions, to size select the target bands. We also sequenced these samples on an Illumina MiSeq platform using a paired-end V2 300 cycle kit. We analyzed all runs together since we did not find substantial run effects that interfered with addressing our hypotheses (S1 Fig).

### Data analysis

Paired-end reads were demultiplexed using Illumina-utils version 2.0.2 [54]. We performed quality filtering, merging of paired reads, and amplicon sequence variant (ASV) clustering using DADA2 version 1.12.1 [55] in R version 3.6.1 [56]. We assigned taxonomy using IDTAXA from the DECPHER package version 2.12.0 [57] with the Silva Small Subunit (SSU) 132 training set for classification. We used the phyloseq package version 1.28.0 in R to perform diversity metric visualizations and statistical tests [58].

The difference between body sites (oral cavity and cloaca) were evaluated using Bray-Curtis distance metrics. We tested for significant differences of Bray-Curtis distance metrics using permutational multivariate analysis of variance (PERMANOVA) for variables including survival, disease condition (Pneumonia vs. Non-Pneumonia), and arrival day at NEAq (day of stranding or next day) for each body site. We performed hierarchical clustering using the simple average method to evaluate the differences between intake samples and convalescent samples (timepoint 4a). Random forest models were used to determine which ASVs were associated with survival (survived vs. died) using the randomForest package version 4.6–14 [59]. To analyze the differences in dispersion between the groups that survived and died, we used the betadisper function and tested for significance with analysis of variance (ANOVA) in R.

Correlation between microbial communities at each body site and clinical parameters were determined using the envfit function with the vegan package version 2.5–6 in R [60]. This function fits vectors representing environmental factors, in this case hematologic values, to ordination plots and tests for statistical significance with 999 random permutation tests. The blood gas, biochemical, and hematologic analytes included pH, partial pressure of carbon dioxide (pCO_2_), partial pressure of oxygen (pO_2_), bicarbonate (HCO_3_), sodium (Na), potassium (K), chloride (Cl), ionized calcium, glucose, blood urea nitrogen (BUN), uric acid, lactate, hematocrit (Hct), white blood cell count, relative heterophil count, and relative lymphocyte count. Uric acid was the only analyte selected from the day 3 chemistry panel due to its relevance to renal function and disease [14,17].

Within a given body site (oral cavity or cloaca), we used principal coordinate analysis (PCoA) of Bray-Curtis distance metrics to visualize variations in the microbial communities across timepoints and days in rehabilitation, and we tested for significant differences between timepoints using pairwise PERMANOVA with p values adjusted for multiple comparisons using the false discovery rate (FDR) method, also known as the Benjamini-Hochberg procedure. Shannon diversity index was calculated for each body site at each timepoint, and significance was tested by pairwise Wilcoxon tests for data not normally distributed or a pairwise Student’s t-test for normally distributed data (S2 Table). A p value of <0.05 was considered statistically significant following application of the Benjamini-Hochberg procedure.

To test the hypothesis that antibiotics affected the microbial communities of turtles and that the microbiome became similar at convalescence to turtles that were not on antibiotics, we calculated Bray-Curtis dissimilarity for the communities at each timepoint. We used pairwise PERMANOVA to determine significant differences based on antibiotic exposures or antibiotic type. A p value of <0.05 was considered statistically significant following application of the Benjamini-Hochberg procedure to correct for multiple comparisons. If there was a significant difference between antibiotic types at a timepoint, we performed a similarity percentages breakdown (SIMPER) analysis [61], to identify abundant ASVs that contribute most to the Bray-Curtis dissimilarity between antibiotic groups. To test whether alpha diversity changed based on antibiotic exposure or drug type, we calculated Shannon diversity, and performed significance testing by pairwise Wilcoxon tests for data not normally distributed or a pairwise Student’s t-test for normally distributed data (S2 Table). A p value of <0.05 was considered statistically significant following application of the Benjamini-Hochberg procedure.

## Results

At intake, we collected oral and cloacal swabs from a total of 35 Kemp’s ridley turtles (Fig 1). Seven turtles died shortly after being admitted to the hospital, and two turtles died later in rehabilitation (after timepoint 2 samples were collected). Overall, 26 turtles had serial samples from intake to convalescence, varying from two to five timepoints depending on clinical status (Fig 1). Of the 35 turtles, veterinarians categorized 15 turtles as Non-Pneumonia and 20 as Pneumonia based on initial radiographs. Five of the turtles categorized as Non-Pneumonia were placed on antibiotics, while the remainder were not. All turtles with pneumonia were placed on antibiotics. Veterinarians did not prescribe antibiotics to 11 turtles (4 of these died) and prescribed antibiotics to 24 turtles (5 of these died), including oxytetracycline (n = 8 surviving turtles) or ceftazidime (n = 11 surviving turtles). Veterinarians prescribed additional antibiotics to five of the turtles that initially received ceftazidime because their disease condition was not improving (typically after one or two months of rehabilitation). These additional antibiotics varied depending on diagnostics, so they are reported as ‘ceftaz, other’ for the purpose of this study. Seven turtles that survived did not receive any systemic antibiotics. Though the median number of days until convalescent samples was 70 days, there was a difference in medians for the turtles on antibiotics and those that were not. Turtles on antibiotics were in rehab for a median of 76 days (range 55 to 201 days) and those not on antibiotics had a median of 50 days (range 24–104 days).

Out of 230 oral and cloacal samples, sequencing of the 16S rRNA gene resulted in 2,309,082 reads after joining paired-end reads and quality filtering, which included the removal of chimeras, singletons, chloroplasts, mitochondrial DNA, and Archaea. The mean sequence counts per sample was 10,039 (median 7,429) and range was 777 to 80,792 counts per sample. These sequences were assigned to 1,528 unique ASVs across 218 different families.

**Fig 2:**
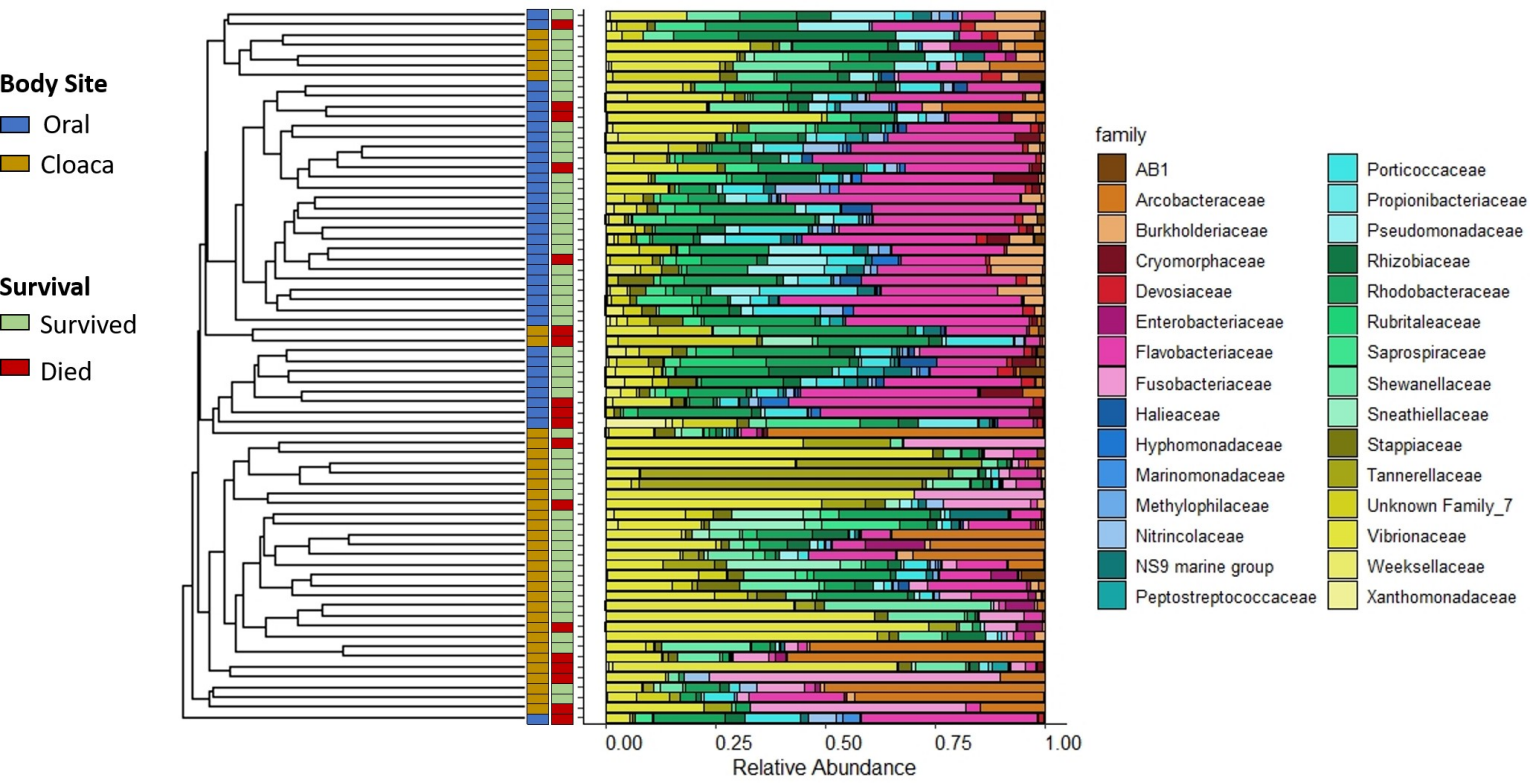
Turtle microbial communities at intake. Hierarchical clustering of intake samples is shown as the dendrogram on the left, with the tips each representing a sample. On the right are the corresponding stacked bar plots representing relative abundance of community composition at the family level (top 30 families). Colored bars at the center specify body site (left bar) and survival (right bar) corresponding to each sample.

Oral samples had significantly higher Shannon diversity than cloacal samples at intake (t-test, p < 0.001; oral mean ± standard deviation 3.45 ± 0.47; cloacal mean ± standard deviation 2.90 ± 0.52), and the oral and cloacal microbiomes were significantly different from each other at intake based on Bray-Curtis dissimilarity (PERMANOVA, p = 0.001), which we visualized via hierarchical clustering of the Bray-Curtis dissimilarity values (Fig 2). The oral microbial communities at intake were dominated by Bacteria in the family Flavobacteriaceae, with a mean abundance of 30.0%, followed by Rhodobacteraceae (13.7%), Vibrionaceae (9.0%), and Porticoccaceae (6.0%). The microbiome of cloacal samples at intake were dominated by Vibrionaceae (23.1%), Arcobacteraceae (11.8%), Shewanellaceae (7.7%), and Rhodobacteraceae (6.7%). There was significant clustering within the oral (PERMANOVA, p = 0.035) and cloacal (PERMANOVA, p = 0.047) microbiomes by survival, based on Bray-Curtis dissimilarity (Fig 2). Other variables that did not contribute to clustering include disease condition (Pneumonia vs. Non-Pneumonia) and arrival day (same day as stranding or next day). There were no significant correlations between blood analytes and microbial communities for either body site at intake.

**Fig 3:**
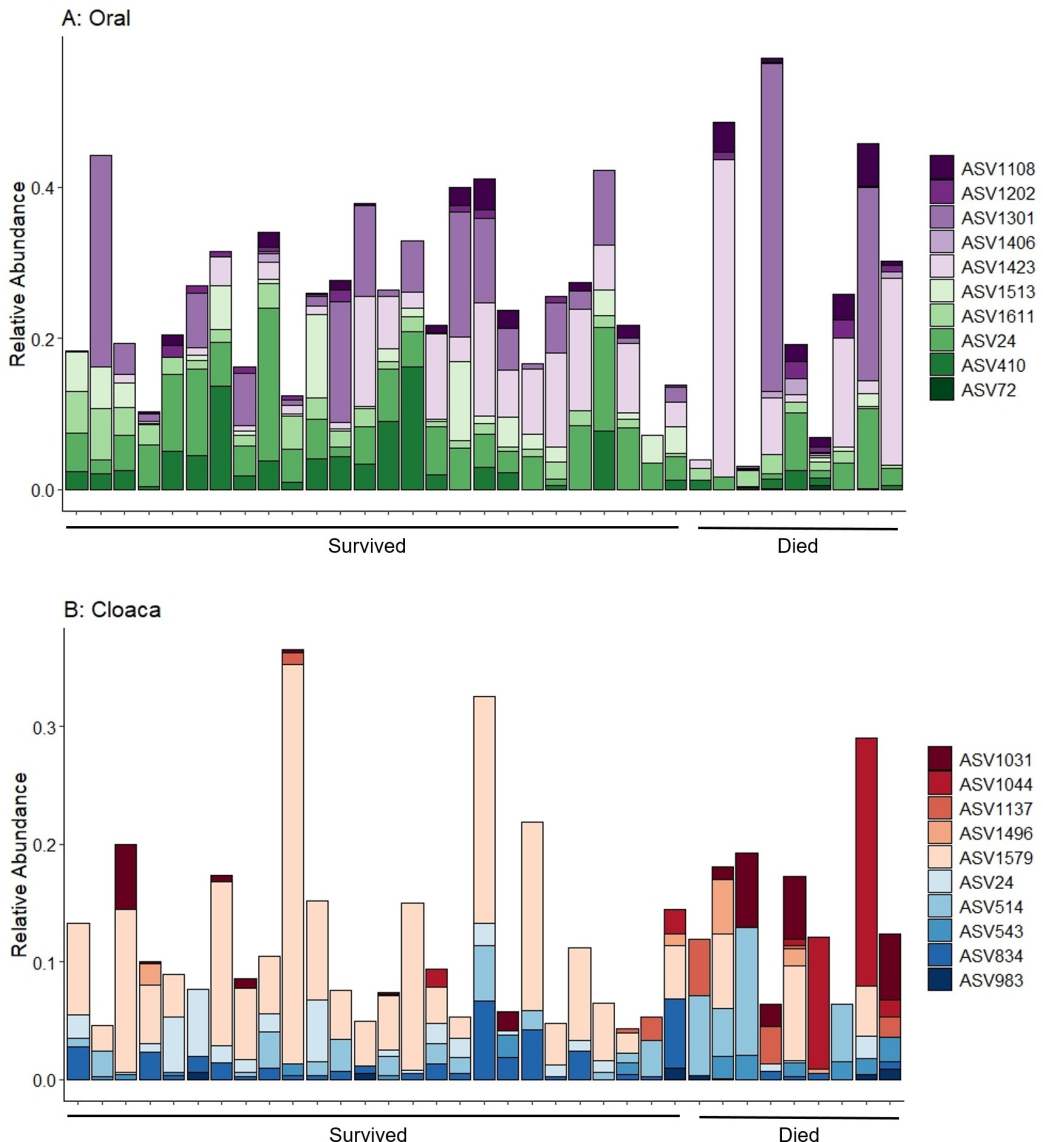
Random forest model predictions for survival. The 10 most significant ASVs at intake that differ between turtles that survived and those that died for oral samples (A) and cloacal samples (B). Taxonomy information for these ASVs is provided in Table 1.

Random forest modelling predicted ASVs that differ between the turtles that survived and those that died for both oral samples (Fig 3A) and cloacal samples (Fig 3B). These analyses had an out-of-bag error rate of 22.9% and 25.7%, respectively. The model correctly predicted turtle survival based on the microbiome from 26 oral samples (100%), however, it correctly predicted turtle mortality in only one oral sample (12%). For cloacal samples, the model correctly predicted turtle survival of 25 cloacal samples (96%), but correctly predicted turtle mortality in only one cloacal sample (12%). Thus, although good at predicting survival, the model struggled to find key indicators that could be used to predict turtle mortality. The top 10 ASVs most important in distinguishing those turtles that survived included taxa from the families Flavobacteriaceae and Rhodobacteraceae for oral samples (Table 1). The oral samples from turtles that died were more variable in the abundance of the important ASVs, but primarily had lower abundance of ASV1513, *Thalassobius* sp., from the family Rhodobacteraceae. For cloacal samples, the top 10 ASVs that differed between those turtles that survived and those that did not included diverse taxa from 9 different families (Table 1). There was high variability in the composition of these ASVs in the turtles that died, with some of the turtles having high proportions of Rhodobacteraceae, Fusobacteriaceae, and Ruminococcaceae, and lower abundance of Burkholderiaceae compared to turtles that survived. Overall, the oral microbiome had greater variance in turtles that died compared to those that survived (ANOVA, p = 0.041), however the difference in variance in the cloacal microbiome of turtles that died was not statistically different than from those that survived (ANOVA, p = 0.074).

**Table 1:** Taxonomy of the top 10 ASVs predicting survival. Survival was predicted by random forest modelling for both oral and cloacal samples at intake.

**ORAL**	**Taxonomy**			
**ASV**	**Class**	**Order**	**Family**	**Genus**
ASV24	Gammaproteobacteria	Cellvibrionales	Porticoccaceae	*Porticoccus*
ASV72	Gammaproteobacteria	Arenicellales	Arenicellaceae	HTCC5015
ASV410	Bacteroidia	Flavobacteriales	Flavobacteriaceae	*Aquimarina*
ASV1108	Alphaproteobacteria	Rhodobacterales	Rhodobacteraceae	NA
ASV1202	Bacteroidia	Flavobacteriales	Flavobacteriaceae	NA
ASV1301	Bacteroidia	Flavobacteriales	Flavobacteriaceae	*Kordia*
ASV1406	Alphaproteobacteria	Rhodobacterales	Rhodobacteraceae	*Pseudophaeobacter*
ASV1423	Gammaproteobacteria	Vibrionales	Vibrionaceae	NA
ASV1513	Alphaproteobacteria	Rhodobacterales	Rhodobacteraceae	NA
ASV1611	Alphaproteobacteria	Rhizobiales	Stappiaceae	NA
**CLOACAL**	**Taxonomy**			
**ASV**	**Class**	**Order**	**Family**	**Genus**
ASV24	Gammaproteobacteria	Cellvibrionales	Porticoccaceae	*Porticoccus*
ASV514	Gammaproteobacteria	Vibrionales	Vibrionaceae	*Photobacterium*
ASV543	Gammaproteobacteria	Alteromonadales	Shewanellaceae	*Shewanella*
ASV834	Gammaproteobacteria	Betaproteobacteriales	Burkholderiaceae	NA
ASV983	Alphaproteobacteria	Rhodospirillales	Rhodospirillaceae	*Candidatus Riegeria*
ASV1031	Fusobacteriia	Fusobacteriales	Fusobacteriaceae	*Cetobacterium*
ASV1044	Alphaproteobacteria	Rhodobacterales	Rhodobacteraceae	NA
ASV1137	Clostridia	Clostridiales	Ruminococcaceae	NA
ASV1496	Campylobacteria	Campylobacterales	Arcobacteraceae	*Arcobacter*
ASV1579	Gammaproteobacteria	Alteromonadales	Shewanellaceae	*Shewanella*

**Fig 4:**
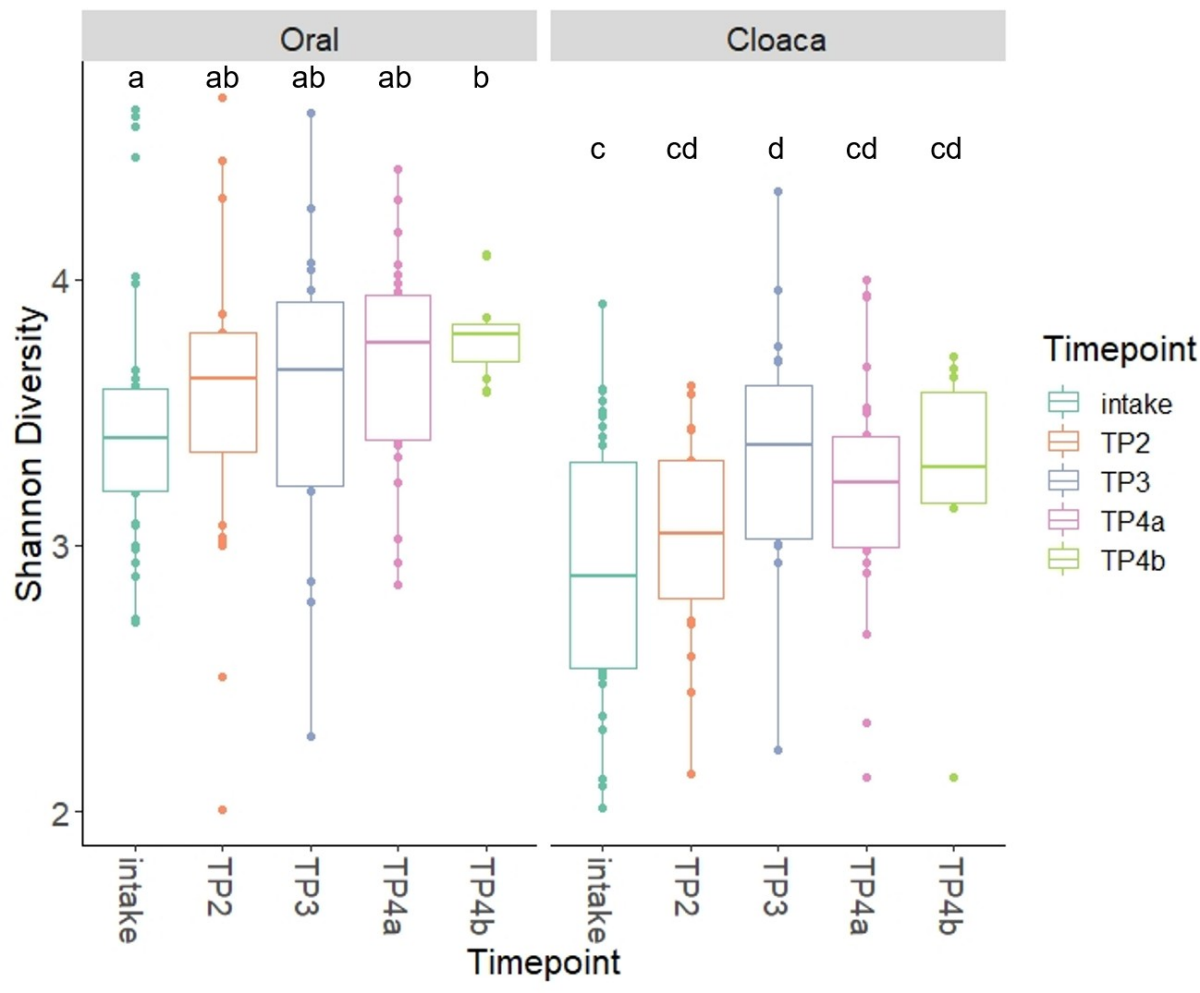
Shannon diversity index of turtles through rehabilitation. Oral samples (left) and cloacal samples (right) at each timepoint during rehabilitation. Different letters above boxplots indicate significant differences in means of Shannon diversity (p < 0.05; S2 Table).

**Fig 5:**
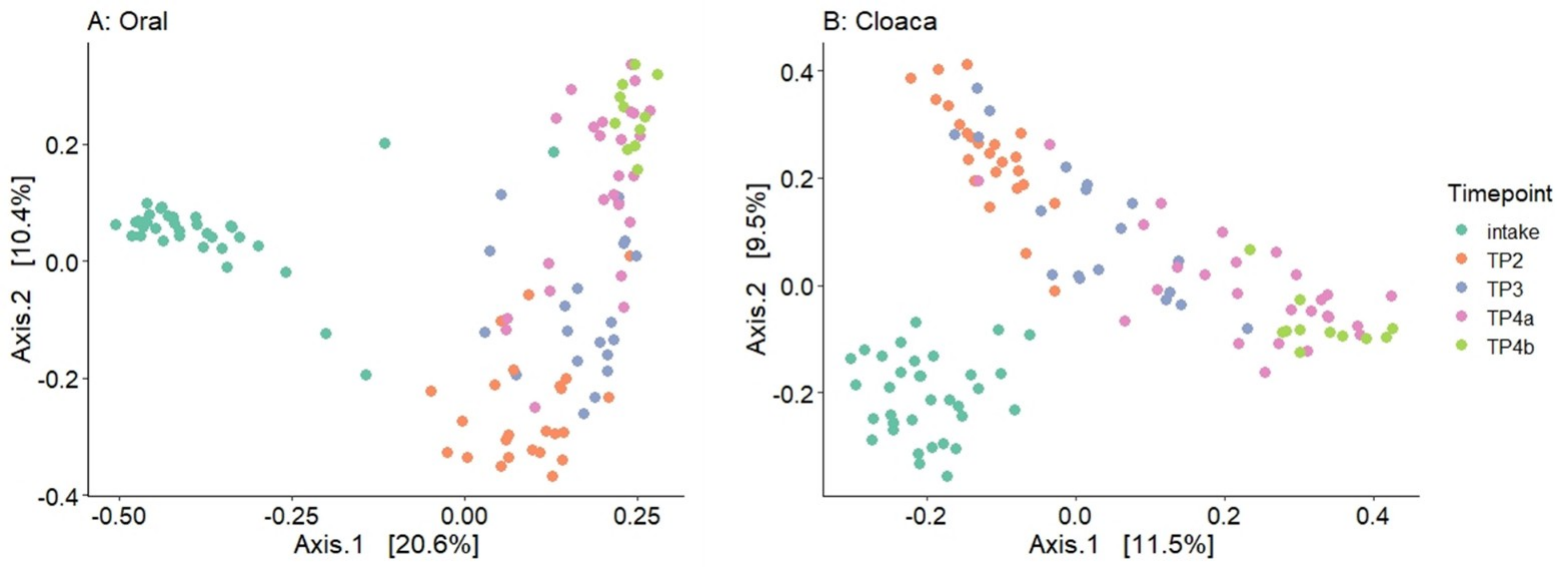
Microbial communities across all timepoints. Principal coordinate analysis (PCoA) plots of oral samples (A) and cloacal samples (B) based on Bray-Curtis distance. Color indicates timepoint in rehabilitation from intake to convalescence (TP4a)/pre-release (TP4b).

**Fig 6:**
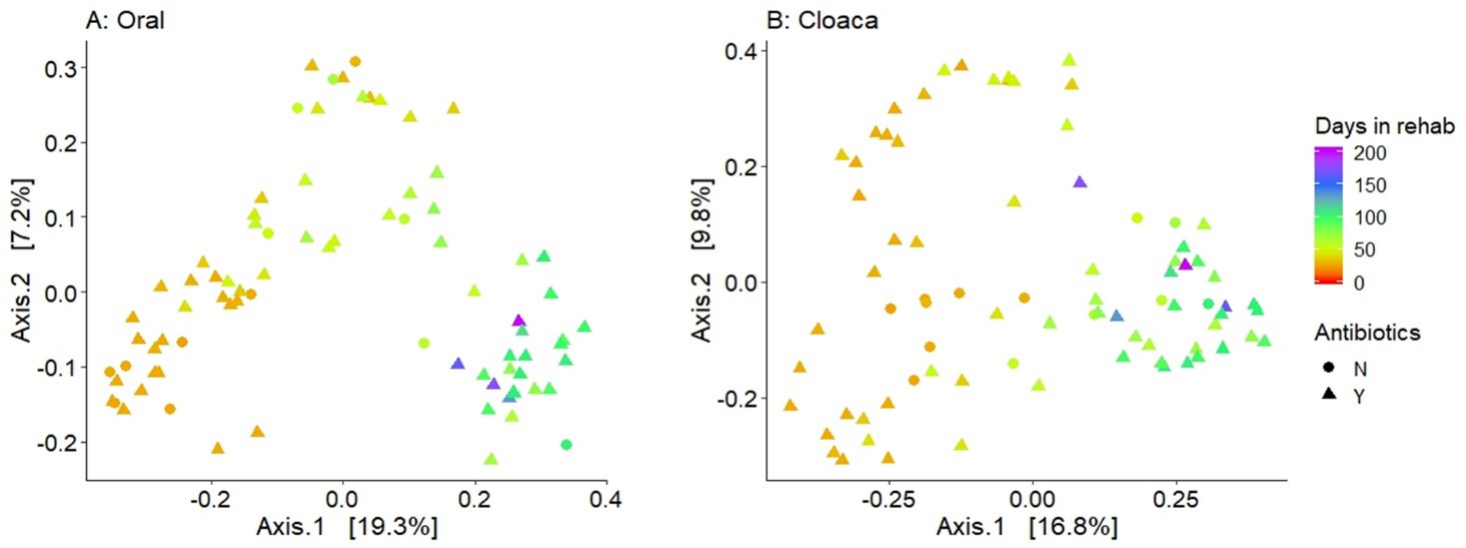
Microbial communities during rehabilitation to convalescence. Principal coordinate analysis (PCoA) plots of oral samples (A) and cloacal samples (B) based on Bray-Curtis distance. Color indicates number of days in rehabilitation. Shape indicates whether a turtle was not on antibiotics (circle, N) or on antibiotics (triangle, Y) during rehabilitation.

There was a slight trend of increasing Shannon diversity from intake to rehabilitation samples for both the oral and cloacal microbiome (Fig 4, S2 Table). We found significant differences between TP4b (pre-release) and intake for oral samples (t-test, p = 0.01), between TP3 (in rehab) and intake for cloacal samples (Wilcoxon, p = 0.028), and between TP3 and TP2 for cloacal samples (Wilcoxon, p = 0.028). The PCoA of each body site for all samples showed significant differences between timepoints based on Bray-Curtis dissimilarity (S3 Table, Fig 5). The largest separation along the principal axis resulted from the difference between intake samples and the remaining samples for both the oral and cloacal communities (Fig 5). After intake, the turtles were in a shared environment at a consistent temperature so we further visualized shifts in microbial communities during rehabilitation by excluding the intake samples (Fig 6). The microbial communities of both oral and cloacal samples continued to shift based on the number of days in rehabilitation. The shift appeared to stabilize after approximately 100 days, with less variability in the microbiome the longer the turtles were in rehabilitation (Fig 6). It is important to note that these shifts during rehabilitation were consistent among turtles that received antibiotics and those that did not.

**Fig 7:**
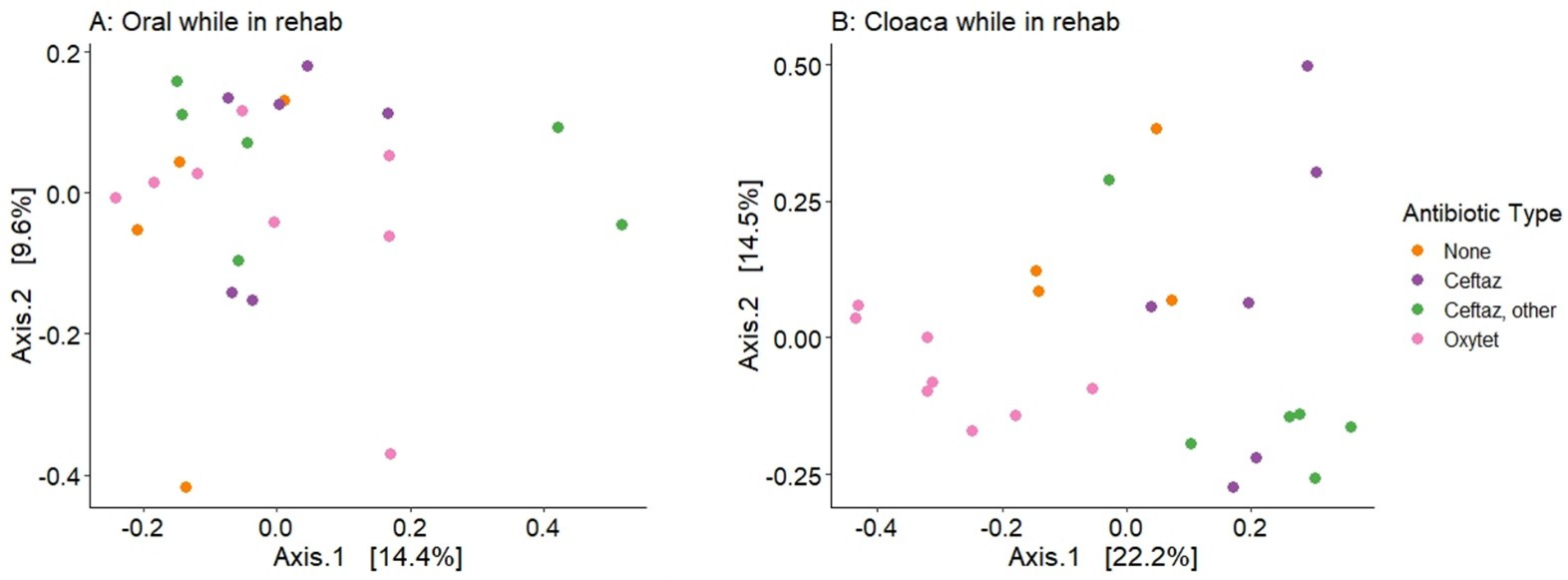
Microbial communities during rehabilitation and antibiotics. Principal coordinate analysis (PCoA) plots of oral (A) and cloacal (B) samples based on Bray-Curtis distance during rehabilitation (TP2). Colors specify the type of antibiotic the turtle was on during hospitalization.

**Fig 8:**
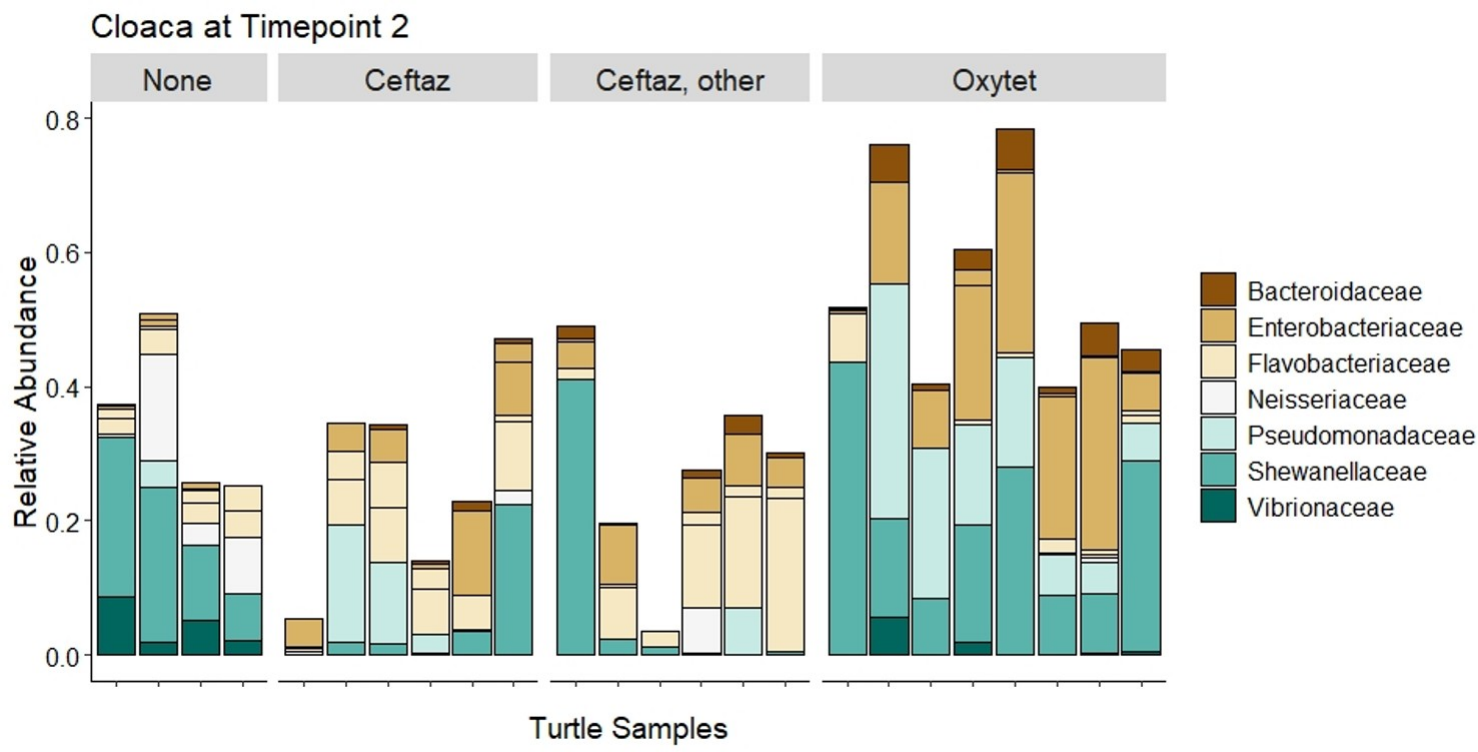
ASVs of cloacal samples at timepoint 2. Relative abundance of ASVs (colored by family) that significantly contribute to the differences between antibiotic type of cloacal samples while in rehab.

We observed changes in turtle microbiomes due to antibiotic exposure at specific timepoints during rehabilitation. At timepoint 2 (approximately four weeks after the start of antibiotic treatment, for those turtles that received antibiotics), there was a significant difference by antibiotic type based on Bray-Curtis distance for cloacal samples, but not for oral samples (Fig 7). Turtles not receiving antibiotics, as well as those on specific antibiotic types (ceftazidime, oxytetracycline, ‘ceftaz, other’), had distinct cloacal microbial communities except for the comparison of Ceftaz vs. ‘Ceftaz, other’ (S3 Table). SIMPER analysis indicated that the families Bacteroidaceae, Enterobacteriaceae, and Pseudomonadaceae were more abundant in cloacal samples of turtles receiving oxytetracycline (Fig 8). Vibrionaceae was more prevalent in the cloacal samples of turtles not receiving antibiotics, while Flavobacteriaceae was more abundant in the cloacal samples of turtles receiving ceftazidime (+/- other). Shewanellaceae was consistently present in the cloaca of turtles that received oxytetracycline and in those that received no antibiotic. There was no significant difference in Shannon diversity based on antibiotic type during rehabilitation for oral samples and cloacal samples.

**Fig 9:**
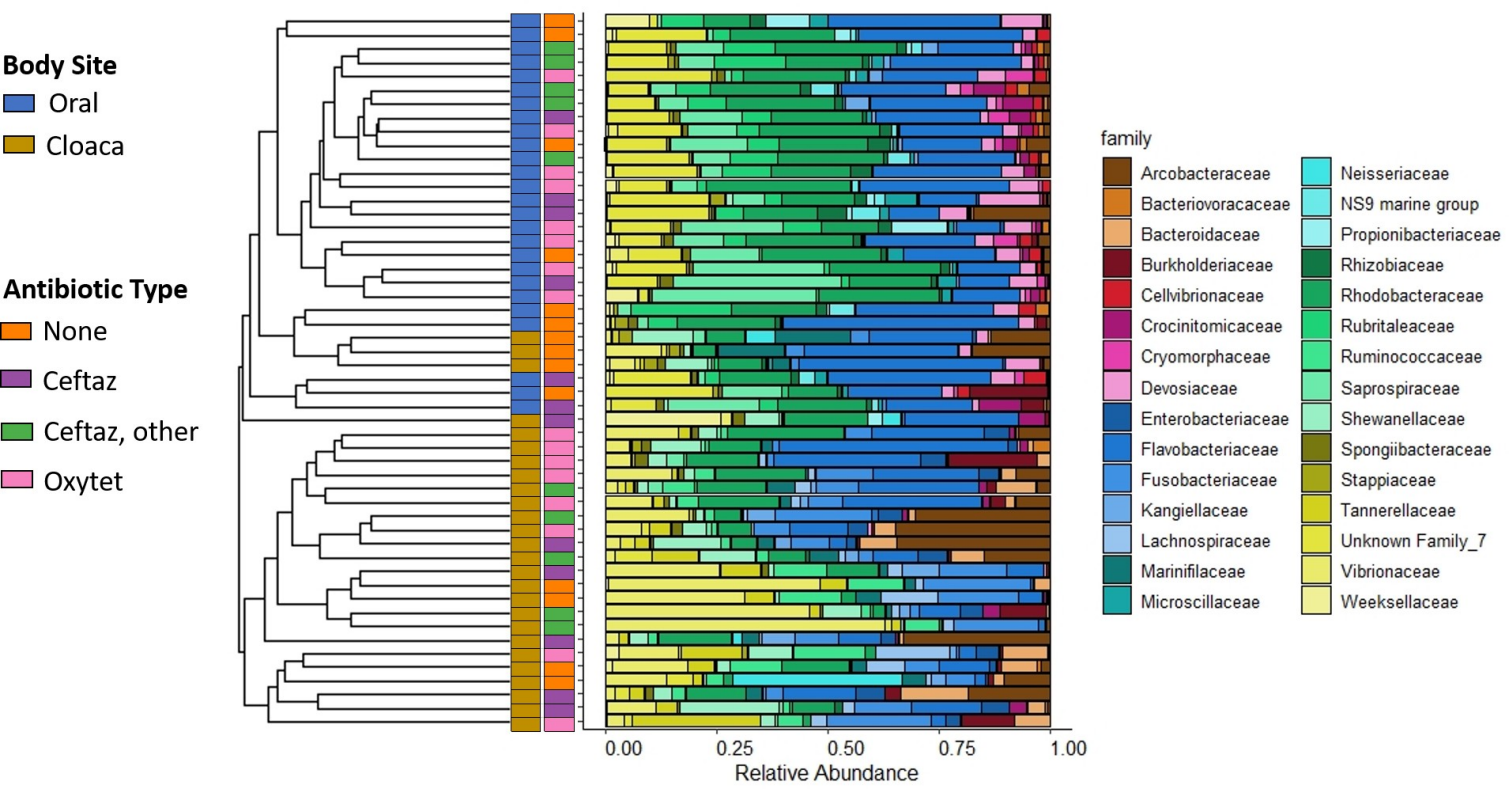
Turtle microbial communities at convalescence. Hierarchical clustering of convalescent samples (timepoint 4a) is shown as the dendrogram on the left, with the tips each representing a sample. On the right are the corresponding stacked bar plots representing relative abundance of community composition at the family level (top 30 families). Colored bars at the center specify body site (left bar) and antibiotic type (right bar) corresponding to each sample.

Bray-Curtis distances at convalescence, defined as 30 days after the antibiotic was discontinued, were significantly affected by administration of ceftazidime in addition to another antibiotic compared to no antibiotics, just ceftazidime, and just oxytetracycline for the oral samples. Only oxytetracycline turtles were significantly different from no antibiotic turtles for cloacal samples at convalescence. (Fig 9, S3 Table). SIMPER analysis indicated that turtles receiving any type of antibiotics had, at convalescence, oral samples with higher abundance of an ASV in the family Microscillaceae (*Microscilla* sp.). Oral samples of convalesced turtles that never received antibiotics had higher prevalence of Saprospiraceae (*Aureispira* sp.), and turtles that received antibiotics in addition to ceftazidime (‘ceftaz, other’) had higher abundances of ASVs specific to the families Flavobacteriaceae (*Maritimimonas* sp.), Rubritaleaceae (*Rubritalea* sp.), and Kangiellaceae (*Aliikangiella* sp.), all of which are marine bacteria. At convalescence, the cloacal samples from turtles that had received antibiotics had an increased presence of the Vibrionaceae *Photobacterium* sp. (more consistent with turtles that never received antibiotics), but the turtles that had other antibiotics in addition to ceftazidime (‘ceftaz, other’) also had some samples with higher abundance of Fusobacteraceae (*Fusobacterium* sp.) at convalescence. All of the ASVs in these families, except for Vibrionaceae, made up a low relative abundance compared to other ASVs in the same families found in the samples collected at convalescence. There was no significant difference in Shannon diversity based on antibiotic type at convalescence for oral samples and cloacal samples.

At convalescence, we also observed that Bray-Curtis distance remained significantly different between oral and cloacal samples (PERMANOVA, p = 0.001, Fig 9). Since convalescent samples were different from intake samples, we also wanted to characterize the differences between body sites prior to release (Fig 9). The oral microbial communities at convalescence were dominated by bacteria in the family Flavobacteriaceae (22.5%) and Rhodobacteraceae (20.6%) followed by an unassigned Gammaproteobacteria family (12.1%) and Saprospiraceae (10.8%). The microbiome of cloacal samples at convalescence were dominated by Flavobacteriaceae (17.0%), Vibrionaceae (13.6%), Arcobacteraceae (10.3%), and Rhodobacteraceae (9.1%). The Shannon diversity of oral microbial communities remained higher compared to the cloacal samples at convalescence (t-test, p < 0.001; oral mean ± standard deviation 3.64 ± 0.39; cloacal mean ± standard deviation 3.19 ± 0.44).

## Discussion

We characterized the oral and cloacal microbiomes of cold-stunned Kemp’s ridley turtles through the course of rehabilitation, from stranding to release. This is the first investigation of the microbiome during rehabilitation after cold stunning for any turtle species. In Kemp’s ridley turtles, the oral and cloacal microbial communities were distinct from each other in composition and taxonomic diversity when they arrived at the rehabilitation center and remained distinct through the course of treatment. The lower Shannon diversity of cloacal samples compared to oral samples is not unique to Kemp’s ridley sea turtles and is thought to be due to greater interaction with transient microbes from the environment that enter the turtle via the oral cavity [62,63].

Compared to wild healthy Kemp’s ridley turtles from the Gulf of Mexico, the oral microbiome of cold-stunned turtles at intake shared similar predominant bacterial families, including Flavobacteraceae and Rhodobacteraceae [64]. The cloacal samples, by contrast, were different between the healthy wild Kemp’s ridley turtles and the cold-stunned stranded turtles with no shared dominant families between the two groups [64]. Cardiobacteraceae, Flavobacteraceae, and Neisseriaceae were most prevalent in the wild healthy turtles, while Vibrionaceae, Arcobacteraceae, Shewanellaceae, and Rhodobacteraceae were prevalent from the intake cloacal samples of cold-stunned turtles. This difference in cloacal samples could be due to diet differences between the regions, last meal for the cold-stunned turtles (which tend to be malnourished due to stranding), temperature, or a more integrated relationship of immune system with gastrointestinal microbes.

Cold-stunned turtles strand with a variety of clinical derangements. Plasma biochemical and hematologic analyses are important for evaluating the health and monitoring the recovery of these animals [5]. We did not find a correlation of blood analytes with the microbiome of either the cloaca or oral cavity of cold-stunned turtles. This was slightly unexpected since blood parameters are useful in diagnosing diseases, metabolic disorders, and immunological disorders that have been linked to the microbiome in other organisms [24,65,66], although flatback turtles (*Natator depressus*) were also found to have no correlation between the microbiome and blood parameters [67]. Some blood analytes of turtles had great variability at intake, which could be one reason for lack of correlation with microbial communities. For example, glucose at admission was highly variable, with hypoglycemia likely indicating exhaustion, anorexia, or sepsis, and hyperglycemia indicating a stress response, liver disease, or pancreatic disease [10,12,68]. White blood cell counts also can be indicative of several conditions including inflammation, immune response, or systemic pathologic conditions [12], but were variable in these turtles at stranding, resulting in no clear association with the microbiome. Blood pH, pCO_2_, pO_2_, and potassium concentrations in particular are good predictors of mortality in cold-stunned Kemp’s ridley turtles [69], yet they were also not associated with the turtle microbiome. It is possible that other factors were overriding the microbiome correlations, such as reduced renal function, dehydration, and sepsis [11–14,17]. Another possibility is that the cloaca is not truly representative of the gut, as there is regional variability along the gastrointestinal tract [75], and thus may be less involved in immune and metabolic disorders. Future studies focusing on adrenal function and specific immune assays of Kemp’s ridley sea turtles, in addition to these traditional blood analytes, and examining other sections of the gastrointestinal tract, might provide further insight into the relationship between the microbiome, health, and the immune system for this species.

Although the clinical parameters and disease conditions did not strongly predict the microbiomes of cold-stunned turtles at admission, we were able to identify ASVs that were different between turtles that survived and those that died. For example, ASV1044, from the genus *Phaebacter*, was found in higher abundance in initial cloacal samples of turtles that died (Fig 3). This genus is an antibiotic producing bacterium that is found in sea water and on marine surfaces [70]. *Phaebacter* sp. strongly shaped the microbiome of microalgae by changing the proportions of other metabolite producing bacteria such as *Vibrio* sp. [71], so this genus could play an important role in altering the sea turtle microbial communities. Cloacal samples of turtles that died also had higher abundance of ASVs in families that are common to marine environments such as Shewanellaceae and Rhodobacteraceae. The oral microbiome of turtles that died were more variable in composition compared to turtles that survived, and random forest models largely failed to detect a diagnostic microbial community associated with the turtles that died. This may be an indication of dysbiosis, in which more diseased animals show greater variability in microbial composition [21,24]. Increased variability in the microbial community has also been associated with coral mortality, in which above average temperatures caused increases in various opportunistic microbes leading to stochastic changes [21]. Although all stranded cold-stunned turtles are not considered healthy, the turtles that died had greater variability in their microbiomes than those that survived. Increased variability of the microbiome of turtles that died could be associated with other events that affect survival, such as aspiration of sea water, resulting in severe pneumonia, respiratory acidosis, and severe electrolyte imbalances [10–12,16,69]. Thus, mortality may be further associated with stochastic changes in the microbial communities rather than to the presence of specific pathogens.

The turtles that survived had microbiomes that shifted through rehabilitation as they stabilized in a controlled environment and recovered from cold-stunning. The local environment shapes distinct microbial communities, as seen in other reptiles and aquatic animals [62,63,72,73]. Shannon diversity was also lower in the intake samples compared to later timepoints in rehabilitation, further suggesting a dysbiosis resulting from cold-stunning (Fig 4). Initially, turtles may remain inappetent for days to weeks after stranding, which could cause differences in the microbiome until they are eating consistently [74,75]. Other reptiles had lower alpha diversity during periods of fasting compared to during the feeding season [74]. Thus, disease recovery and feeding status may be variables that further shift the oral and cloacal microbiome during rehabilitation. There are also changes in the number of turtles in the tanks during the first few months of rehabilitation, which influences the bioload of the system, adding additional variables that could influence microbial community composition. During the first few months of rehabilitation, cold stunned turtles are in various states of disease and drug exposure. It may take several months before turtles recover from cold-stunning, and this appears to apply to their microbiome as well.

In addition to microbiome changes over time, we found changes due to antibiotic exposure, but not as many changes as might be expected based on what is known about the effect of antibiotics in other animals. For example, we did not see the typical reduction in Shannon diversity of turtles on antibiotics versus those not on antibiotics [38,41], but we did find differences in beta diversity (Fig 7), which was a similar finding to rehabilitating Kemp’s ridley turtles that were incidentally captured in Mississippi [35]. The ASVs that were more abundant in cloacal samples of turtles receiving oxytetracycline were from the families Pseudomonadaceae, Vibrionaceae, and Enterobacteriaceae. Although these are all bacterial families targeted by the drug, antibiotic resistance and transient microbes continuously passing through the gastrointestinal tract cause them to remain present in the microbiome. We may be capturing transient microbes that are constantly being introduced by the local environment and food, thus antibiotics would not affect their presence in the samples collected. Ceftazidime and oxytetracycline are so commonly used in sea turtles that antibiotic resistance is a concern at rehabilitation facilities [37]. Although antibiotic resistance was outside the scope of what we investigated, bacteria in the families Pseudomonadaceae and Enterobacteriaceae have shown high resistance to tetracycline classes of antibiotics, such as oxytetracycline, in sea turtles [76,77]. There were also some strains that were resistant to ceftazidime, though the majority were susceptible in loggerhead turtle cultures [77]. In addition to some bacterial families that were present despite being targets of the antibiotics, several ASVs were reduced in the cloacal samples of turtles that received antibiotics compared to those that did not, revealing that the antibiotics were having an influence despite transient microbes and potential antibiotic resistance. Vibrionaceae, specifically the ASV matching to *Photobacterium damselae*, which is a marine bacterium capable of causing infection in animals [78], was more prevalent in turtles that did not receive antibiotics. *Shewanellae algae* was identified as the ASV in the Shewanellaceae family that had lower abundance in the cloaca of turtles that received ceftazidime, showing the effect these antibiotics have on a variety of bacteria.

During rehabilitation, antibiotics did not affect the oral microbiome as much as they affected the cloacal microbiome. Different body sites may be affected by antibiotics in different ways. The salivary microbiome of humans was not affected by antibiotics although fecal microbial communities were highly affected [43]. Route of administration (injectable or oral) may also play a role, especially depending on drug excretion routes [79]. Drugs such as ceftazidime and oxytetracycline are excreted through the kidneys [37], therefore there may be minimal drug exposure of the oral cavity if delivered by injection [79].

At convalescence, the oral and cloacal microbiomes were both different based on antibiotic type (Fig 9), primarily when comparing ‘ceftaz, other’ to other antibiotics for oral samples and comparing oxytetracycline to no antibiotics for cloacal samples. Although there are no analogous studies in turtles, in humans, throat and gut microbiomes were also both perturbed by antibiotics, but each subject responded uniquely [41]. The time of recovery to a pre-treatment state varied as well, ranging from weeks to several years in humans [41]. The length of time in rehabilitation may also be influencing differences in the microbiome for turtles that were on antibiotics versus those that were not. In several instances, the turtles not on antibiotics were considered convalescent earlier than those on antibiotics. The longer a turtle is in rehabilitation, the likelihood that the environment, including diet, continues to influence shifts in the microbiome and should be considered.

There were several differences in composition between convalescent microbial communities and intake samples for each body site of turtles. Flavobacteriaceae became the most abundant bacterial family of the cloaca at convalescence and remained the most abundant in oral samples. Oral samples also had higher proportions of the marine environmental bacteria Saprospiraceae and an unknown family of Gammaproteobacteria at convalescence. Differences from intake cloacal samples to convalescence were seen in green turtles as well, and the presence of *Salmonella* in convalescent samples specifically indicated introduction from the hospital tanks [29]. We did not see *Salmonella* in our samples, although we did have the closely related *Citrobacter* sp., which is also a coliform bacterium from the family Enterobacteraceae, but this was present in low abundance in both the intake and convalescent samples indicating the hospital environment was not the primary source for this family of bacteria. In addition to the local environment, captivity also plays a role in altering microbial communities due to diet, so mimicking the wild is important to maintaining proper functioning upon release [80,81]. During rehabilitation, Kemp’s ridley turtles are fed high calorie herring and squid compared to the crustaceans they eat in the wild. Shifting the diet to crustaceans during rehabilitation may aid in restoring normal cloacal microbial communities, as was suggested for green turtles transitioning to an herbivorous diet as soon as possible in rehabilitation [34]. However, dietary adjustments away from existing successful protocols should be considered carefully, as items that are less easily digested may cause gastrointestinal disorders in compromised patients [82]. Further evaluating diet as well as the functional microbiome might be a useful method of future studies to determine differences between wild healthy turtles and captive turtles prior to release.

## Conclusions

We characterized the oral and cloacal microbiome of cold-stunned Kemp’s ridley turtles throughout rehabilitation, allowing us to investigate differences in microbial communities based on survival and disease condition. We sampled at multiple timepoints in rehabilitation, from admission to the hospital, during rehabilitation, and at convalescence, providing first glimpses into the changes that occur during recovery from cold-stunning. We identified ASVs that are important to predicting survival of turtles after stranding. An important contributing variable to microbial communities is exposure to antibiotics, which we investigated as well and found that antibiotics did lead to an altered state. Our findings indicate that the microbiome of cold-stunned Kemp’s ridley turtles is affected by disease status, the local environment, and antibiotics, all of which ultimately play a role in the recovery and release status of the turtles. Investigating the functional microbiome and additional clinical parameters from cold-stunning, such as adrenal function, blood cultures, and specific immune assays, might also provide further insight into microbial communities of Kemp’s ridley turtles.

## Supporting information

S1 Fig Microbial communities across all timepoints and sequencing runs. Principal coordinate analysis (PCoA) plots of oral samples (A) and cloacal samples (B) based on Bray-Curtis distance. Color indicates timepoint in rehabilitation from intake to convalescence (TP4a)/pre-release (TP4b). Shape indicates the sequencing run of the sample. Runs 1 and 2 were done using the first purification method, and Run 3 was done using the second purification method described in Materials and methods section.
(PDF)

S1 Table Blood gas, biochemical, and hematologic analytes for each turtle. Subscript TC (temperature corrected) and subscript cor (corrected) indicates manually corrected values based on standard equations (see materials and methods).
(XLSX)

S2 Table Comparison between Shannon diversity of groups. A p value of <0.05 is significant. The statistical test is specified to identify which groups were normally distributed with a pairwise t-test used for normally distributed data (Shapiro-Wilks p > 0.05) and a pairwise wilcoxon test used for data not normally distributed (Shapiro-Wilks p < 0.05).
(XLSX)

S3 Table Results of pairwise PERMANOVA results based on Bray-Curtis dissimilarity. A p value of <0.05 is significant.
(XLSX)
