# Supplementary material for: Characterization of oral and cloacal microbial communities in cold-stunned Kemp’s ridley sea turtles (Lepidochelys kempii) during the time course of rehabilitation
Source: PLoS One. 2021 May 27;16(5):e0252086. doi: 10.1371/journal.pone.0252086 (PMC8159006; doi:10.1371/journal.pone.0252086)
Supplement: S1 Fig — Principal coordinate analysis (PCoA) plots of oral samples (A) and cloacal samples (B) based on Bray-Curtis distance. Color indicates timepoint in rehabilitation from intake to convalescence (TP4a)/pre-release (TP4b). Shape indicates the sequencing run of the sample. Runs 1 and 2 were done using the first purification method, and Run 3 was done using the second purification method described in Materials and methods section. (PDF) [file pone.0252086.s001.pdf]

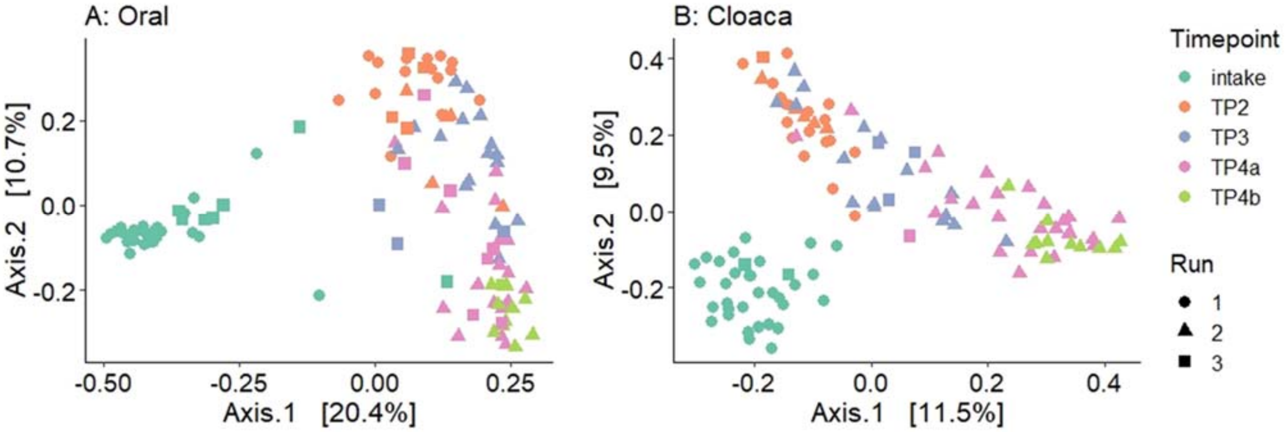

S1 Fig. Microbial communities across all timepoints and sequencing runs. Principal coordinate analysis (PCoA) plots of oral samples (A) and cloacal samples (B) based on Bray-Curtis distance. Color indicates timepoint in rehabilitation from intake to convalescence (TP4a)/pre-release (TP4b). Shape indicates the sequencing run of the sample. Runs 1 and 2 were done using the first purification method, and Run 3 was done using the second purification method described in Materials and methods section.
